# Synergistic cytotoxic activity of cannabinoids from *cannabis sativa* against cutaneous T-cell lymphoma (CTCL) *in-vitro* and *ex-vivo*

**DOI:** 10.18632/oncotarget.27528

**Published:** 2020-03-31

**Authors:** Moran Mazuz, Amir Tiroler, Lilach Moyal, Emmilia Hodak, Stalin Nadarajan, Ajjampura C. Vinayaka, Batia Gorovitz-Haris, Ido Lubin, Avi Drori, Guy Drori, Owen Van Cauwenberghe, Adi Faigenboim, Dvora Namdar, Iris Amitay-Laish, Hinanit Koltai

**Affiliations:** ^1^Agricultural Research Organization (ARO), Volcani Center, Rishon LeZion, Israel; ^2^The Mina and Everard Goodman Faculty of Life Sciences, Bar-Ilan University, Ramat Gan, Israel; ^3^Division of Dermatology, Rabin Medical Center, Petach Tikva, and Sackler Faculty of Medicine, Tel Aviv University, Tel Aviv, Israel; ^4^Laboratory for Molecular Dermatology, Felsenstein Medical Research Center, Rabin Medical Center, Petach Tikva, and Sackler Faculty of Medicine, Tel Aviv University, Tel Aviv, Israel; ^5^Core Facility, Felsenstein Medical Research Center, Rabin Medical Center, Petach Tikva, and Sackler Faculty of Medicine, Tel Aviv University, Tel Aviv, Israel; ^6^MedC Biopharma Corporation, Ontario, Canada; ^7^AgMedica Bioscience Inc., Chatham-Kent, Canada; ^*^These authors equally contributed as the first author; ^#^These authors equally contributed as the last author

**Keywords:** cutaneous T-cell lymphoma, cannabis, cannabinoids, peripheral blood lymphocytes, CTCL cell lines

## Abstract

*Cannabis sativa* produces hundreds of phytocannabinoids and terpenes. Mycosis fungoides (MF) is the most common type of cutaneous T-cell lymphoma (CTCL), characterized by patches, plaques and tumors. Sézary is a leukemic stage of CTCL presenting with erythroderma and the presence of neoplastic Sézary T-cells in peripheral blood. This study aimed to identify active compounds from whole cannabis extracts and their synergistic mixtures, and to assess respective cytotoxic activity against CTCL cells. Ethanol extracts of *C. sativa* were analyzed by high-performance liquid chromatography (HPLC) and gas chromatography/mass spectrometry (GC/MS). Cytotoxic activity was determined using the XTT assay on My-La and HuT-78 cell lines as well as peripheral blood lymphocytes from Sézary patients (SPBL). Annexin V assay and fluorescence-activated cell sorting (FACS) were used to determine apoptosis and cell cycle. RNA sequencing and quantitative PCR were used to determine gene expression. Active cannabis compounds presenting high cytotoxic activity on My-La and HuT-78 cell lines were identified in crude extract fractions designated S4 and S5, and their synergistic mixture was specified. This mixture induced cell cycle arrest and cell apoptosis; a relatively selective apoptosis was also recorded on the malignant CD4^+^CD26^-^ SPBL cells. Significant cytotoxic activity of the corresponding mixture of pure phytocannabinoids further verified genuine interaction between S4 and S5. The gene expression profile was distinct in My-La and HuT-78 cells treated with the S4 and S5 synergistic mixture. We suggest that specifying formulations of synergistic active cannabis compounds and unraveling their modes of action may lead to new cannabis-based therapies.

## INTRODUCTION

*Cannabis sativa* has been used by humanity for thousands of years. Initial interest in the plant was likely related to its psychotropic effects [1]. These effects are mostly due to ∆^9^-tetrahydrocannabinol (THC), the decarboxylated form of ∆^9^-tetrahydrocannabinolic acid (THCA), one of the many phytocannabinoids produced by the plant. Another widely studied phytocannabinoid is non-psychoactive cannabidiol (CBD), a decarboxylated form of cannabidiolic acid (CBDA) [2]. Almost 200 other phytocannabinoids are known in cannabis [3], and more than 160 terpenophenolic compounds have been identified [4]. Many other compounds are also produced in the plant, including alkaloids and flavonoids [5].

THC (mainly ∆^9^-THC and its isomer ∆^8^-THC) is known to activate the endocannabinoid receptors CB_1_ and CB_2_ [3, 6]. CB_1_ and CB_2_ are G-protein coupled receptors that mediate the synaptic and cellular effects of endocannabinoids in various cells and tissues [7]. CB receptors are also present in various cell types in the skin (e. g., [8]), and are expressed in T-lymphocytes [9, 10].

Cutaneous T-cell lymphomas (CTCLs) encompass a heterogeneous group of non-Hodgkin lymphomas [11]. Mycosis fungoides (MF) is the most common CTCL (accounting for 60% of CTCL patients). In its earlier stages it presents as skin lesions, including patches and/or plaques. At advanced stages of disease, patients may suffer from tumors or confluence of erythema that covers ≥ 80% of the surface of their skin (erythroderma). In addition, they may develop involvement of the blood and/or lymph nodes and/or viscera in the disease. Sézary syndrome is a rare type of CTCL in which malignant cells circulate in peripheral blood, also referred to as the leukemic phase of erythrodermic CTCLs. Accounting for only ~3% of cases, these patients have generally poor prognoses [12].

The goal of treating MF and Sézary syndrome is to minimize symptomatic morbidity, preserve quality of life, and to limit disease progression. Most common skin-directed therapies include topical corticosteroids, nitrogen mustard (mechlorethamine), phototherapy, and radiotherapy. The main systemic treatments include interferon-α, oral bexarotene or other retinoids, extracorporeal photopheresis, antifolates (methotrexate, pralatrexate), histone deacetylase inhibitors such as vorinostat and romidepsin, alemtuzumab, liposomal doxorubicin, gemcitabine and the new agents brentuximab vedotin and mogamulizumab [12, 13].

Various phytocannabinoids exhibit antitumor effects in a wide array of cell lines and animal models [14, 15]. On T-cell leukemia cell lines, combinations of THC and CBD, as well as CBD and cannabigerolic acid (CBGA), were found to elicit cell death when each phytocannabinoid was used alone or in combination with each other. In addition, THC and/or CBD enhanced anti-leukemia chemotherapy activity *in vitro* [16, 17]. However, the effect of pure cannabinoids or cannabis extracts on CTCLs is unknown. In addition, despite accumulating knowledge regarding the anti-cancer activity of phytocannabinoids, CB agonists and antagonists, little is known of anti-cancer activity resulting from mixtures of compounds from whole cannabis plant extracts. This may be significant, as in some cases the unrefined content of cannabis inflorescence is superior to isolated compounds [18].

In this paper we identify active compounds derived from *C. sativa* whole plant extracts and their synergistic mixtures, which show cytotoxic activity on CTCL cell lines. This combination of compounds was also active on malignant enriched cells of peripheral blood lymphocytes from Sézary patients (SPBL). The mode of action of the cannabis-derived compounds was partially unraveled based on gene expression profiles.

## RESULTS

### High CBD cannabis strain extract and fractions of this extract show dose dependent cytotoxic activity against My-La cells

**Figure 1:**
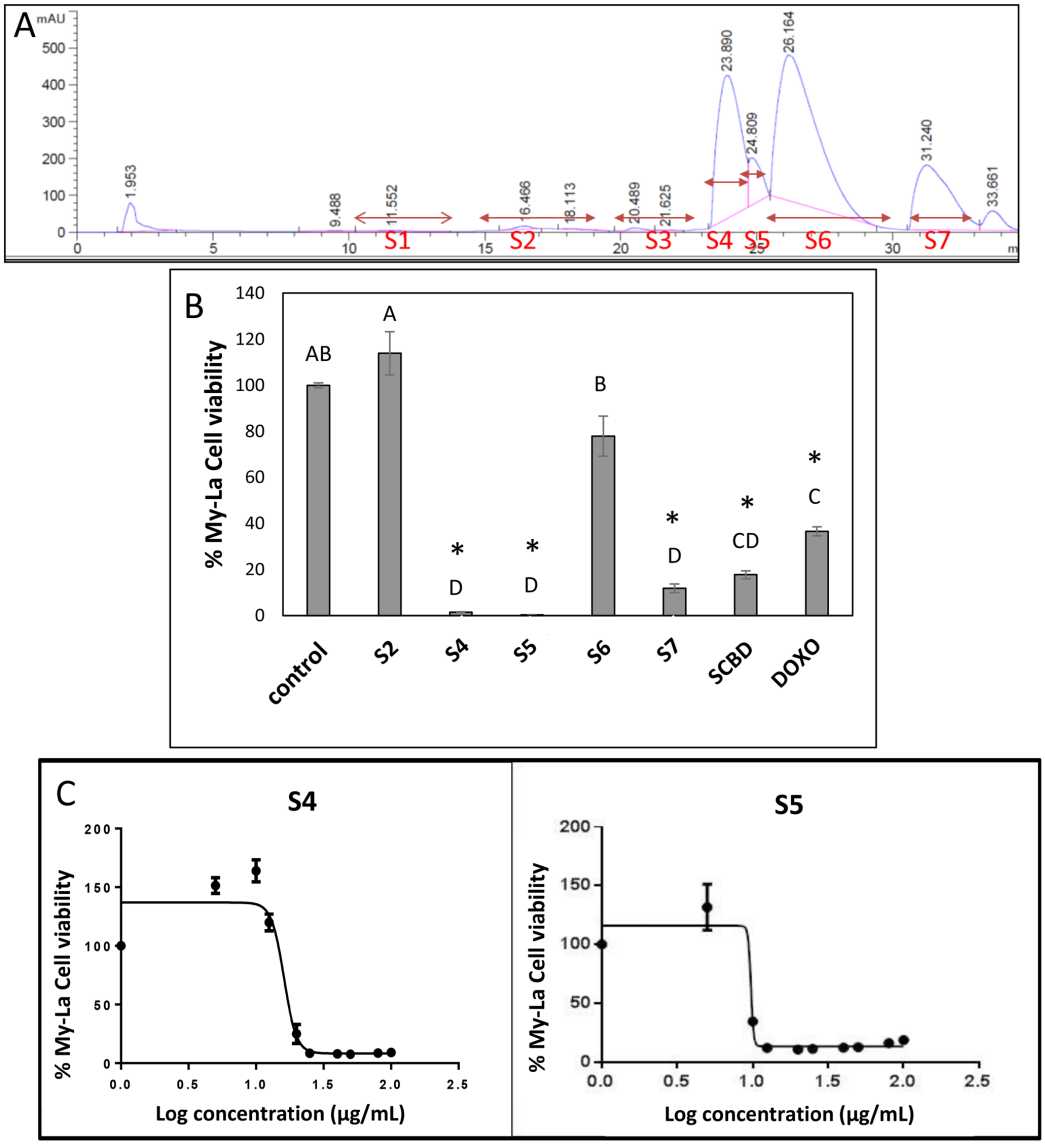
(**A**) HPLC profile of fractions of *C. sativa* SCBD extract. HPLC profile was obtained from preparative HPLC. Fractions were collected as indicated in the Figure. S2, S4, S5, S6 and S7 represent the five fractions into which the peaks were divided. (**B**) Cell viability of My-La cells treated with different fractions of *C. sativa* SCBD extracts. Cell viability was determined by XTT assay as a function of live cell number. Cells were seeded and treated with *C. sativa* ethanol extracts of SCBD (crude), S2, S4, S5, S6 and S7 at a concentration of 40 μg/mL for 48 h. Doxorubicin (DOXO, 300 nM) served as a positive control. Methanol (control) treatment served as solvent control. Values were calculated as the percentage of live cells relative to the solvent control after reducing the absorbance without cells. Error bars indicate ± SE (*n* = 3). Levels with different letters are significantly different from all combinations of pairs by Tukey–Kramer honest significant difference (HSD; *P* ≤ 0.05). ^*^indicates significantly different mean from the control based on Student *T*-test (*P* ≤ 0.05). (**C**) Dose-effect curves of fractions S4 or S5 of *C. sativa* SCBD extract on the viability of the My-La cell line. Data points were connected by non-linear regression lines of the sigmoidal dose-response relation. GraphPad Prism was used to produce dose-response curve and IC50 doses for S4 and S5 fractions.

Ethanol extract of a high-CBD strain of cannabis, SCBD (International Medical Cannabis, IMC, Israel) was cytotoxic to the My-La (MF) cell line, with a calculated IC50 of 25.35 μg/mL following 48 h of treatment. The SCBD extract was fractionated and 5 of the fractions that included HPLC-detected compounds were examined for cytotoxic activity (Figure 1A). Following 48 h of treatment, fractions S4 and S5 were found to have high cytotoxic activity at 40 μg/mL (a relatively high concentration initially selected based on [19] to differentiate active from non-active fractions). Activity of S4 or S5 was ~12- and ~44-fold greater, respectively, than SCBD crude extract at 40 μg/mL and higher than doxorubicin at a concentration of 300 nM (Figure 1B). The cytotoxic activity of S4 and S5 was found to be dose dependent, with the IC50 of S4 at 16.09 μg/mL (Figure 1C) and that of S5 at 9.72 μg/mL (Figure 1C). Fractions S2 and S6 did not show significant cytotoxic activity on the My-La cell line, whereas S7 had reduced cytotoxic activity in comparison to S4 and S5. Methanol at concentrations used to dissolve the treatments (control) had no effect on cell viability (Figure 1B).

### Fractions S4 and S5 have synergistic interactions for cytotoxic activity on My-La and are highly active on HuT-78 cells

The combination of S4 and S5 fractions were examined at various concentrations. Using the Bliss model calculation, several combinations of S4+S5 showed synergistic cytotoxic activity on My-La cells following 48 h of treatment (Table 1). Importantly, combinations of S4+S5 that were found to be significantly cytotoxic to My-La were also highly cytotoxic to the HuT-78 (Sézary) cell line following 48 h of treatment (Table 1). Combinations of the S4+S5 fractions at lower concentrations (e. g., S4 [5 μg/mL] + S5 [2 μg/mL]) showed reduced activity on the My-La and HuT-78 cell lines (Table 1). The compositions of S4 and S5 were determined based on GC/MS and HPLC analysis. S4 contained mainly CBD (98.3%), as well as small proportions of THC (0.3%), CBG (0.2%), a-bisabolol (0.9%) and a minute amount of CBDV (0.09%). S5 contained mainly CBG (58.8%) and CBD (38.2%), as well as small proportions of THC (0.7%) and CBC (0.4%) (Supplementary Figure 1).

**Table 1:** Cell viability of My-La, HuT-78 cells and peripheral blood lymphocytes of Sézary patients (SPBL) treated with S4+S5, and synergy calculations of cytotoxic activity based on the bliss model of S4 and S5 combinations on My-La cells

Treatment	% cell viability in My-La	% cell viability in HuT-78	Bliss model calculated value	Bliss model experimental value	Synergy on My-La cells	% cell viability in SPBL
S4 20 **μg/mL** + S5 10 **μg/mL**	6.09 ± 1.51	15.74 ± 1.02	13.78	9.73	+	20.32 ± 7.06 (*n* = 2)
S4 5 **μg/mL** + S5 6 **μg/mL**	37.10 ± 7.72	10.38 ± 1.55	170.30	22.24	+	55.98 ± 4.37 (*n* = 7)
S4 5 **μg/mL** + S5 2 **μg/mL**	122.19 ± 6.91	97.79 ± 7.26	121.04	133.55	—	80.92 ± 8.69 (*n* = 4)
S4 4 **μg/mL** + S5 4 **μg/mL**	68.86 ± 11.24	49.34 ± 3.44	166.89	62.67	+	ND

Determination of cell viability using XTT assay as a function of live cell number. Cells were treated for 48 h with S4+S5 at different concentrations. Values were calculated as the percentage of live cells relative to the non-treated control. ND: not determined. + indicates synergy.

### Activity of pure phytocannabinoids on My-La and HuT-78 cell lines

**Figure 2:**
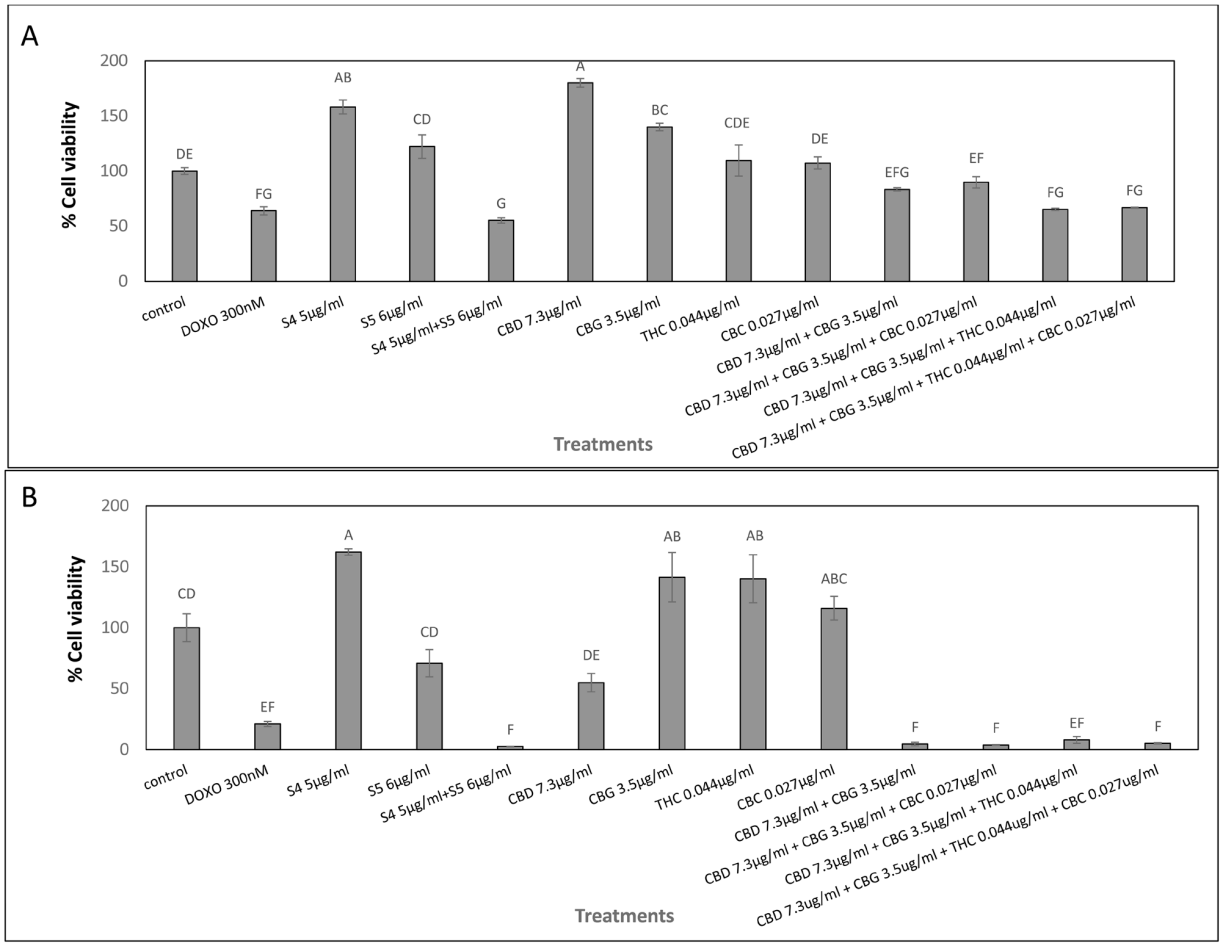
Cell viability of My-La (**A**) and HuT-78 (**B**) cells treated with synergistic concentrations of S4, S5, S4+S5 fractions and with pure CBD, CBG, THC and CBC. Determination of cell viability using XTT assay as a function of live cell number. Cells were seeded and treated with S4 (5 μg/mL), S5 (6 μg/mL) or S4 (5 μg/mL) + S5 (6 μg/mL), CBD (7.3 μg/mL), CBG (3.5 μg/mL), THC (0.044 μg/mL) and CBC (0.027 μg/mL), or a combination of these pure compounds, for 48 h. Methanol (control) treatment served as solvent control. Doxorubicin (DOXO, 300 nM) served as positive control for cell cytotoxicity. Error bars indicate ± SE (*n* = 3). Levels with different letters are significantly different from all combinations of pairs treated by a certain S4+S5 combinations or untreated, according to Tukey-Kramer honest significant difference (HSD; *P* ≤ 0.05).

The cytotoxic activity of the main phytocannabinoids found in S4+S5 mixture—as purified form and in concentrations present in the S4+S5 mixture—was examined. Each phytocannabinoid was examined separately for activity, followed by an examination of the cytotoxic effect of their combinations (Figure 2). The results demonstrated that in My-La, pure CBD at the concentration (7.3 μg/mL) equal to those found in the S4 (5 μg/mL) + S5 (6 μg/mL) mixture was not sufficient to produce cell death. Rather, it led to cell proliferation (Figure 2A). However once CBD was combined with CBG at the concentration present in S4+S5 mixture (3.5 μg/mL) and other phytocannabinoids found in minor amounts in this mixture, i. e., CBD+CBG+THC or CBD+CBG+THC+CBC, cytotoxicity was increased (Figure 2A).

In HuT-78 cells, CBD (7.3 μg/mL) equal to that found in S4 (5 μg/mL) + S5 (6 μg/mL) treatment was potent and led to ~50% cell death. However, similar to the effect on My-La cells, only combinations of CBD+CBG, CBD+CBG+CBC, CBD+CBG+THC or CBD+CBG+THC+CBC, showed cytotoxic activity similar to that of the S4+S5 treatment (Figure 2B).

### CB_2_ expression pattern in My-La cell line or PBL does not correspond to its inverse agonist activity

My-La and HuT-78 cells were treated with a mixture of S4 (5 μg/mL) + S5 (6 μg/mL) with different concentrations of CB_1_ (AM251) or CB_2_ (SR144528) inverse agonists (IA). Treatment of My-La and HuT-78 cells with CB_1_ IA at 1, 5 or 10 μM did not change cytotoxicity of S4+S5 treatment. CB_2_ IA application at 10 μM only decreased 4-fold the cytotoxic activity of S4+S5 treatment in My-La cells only.

However, CB_2_ (gene ID 1269; CNR2) was basally expressed in HuT-78 cells but not in the My-La cell line. This expression pattern was almost unchanged with S4 or S5 treatments and slightly and insignificantly increased in HuT-78 by S4+S5 treatment (Supplementary Table 1). CB_1_ (gene ID 1268; CNR1) was basally expressed in My-La cells but not in HuT-78 cells, and this expression was slightly and insignificantly reduced by S4 treatment and slightly and insignificantly increased by S5 treatment. In comparison, S4+S5 treatment led to a significant increase in CB_1_ expression in My-La cells (Supplementary Table 1). Relative expression levels of the CB receptors determined by quantitative PCR differed between SPBL in comparison to PBL of healthy individuals (HPBL); CB_1_ and CB_2_ were overexpressed in some Sézary patients SPBL yet reduced in others (Supplementary Table 2).

### S4+S5 treatment induces cell cycle arrest and cell apoptosis in My-La and HuT-78 cell lines

**Figure 3:**
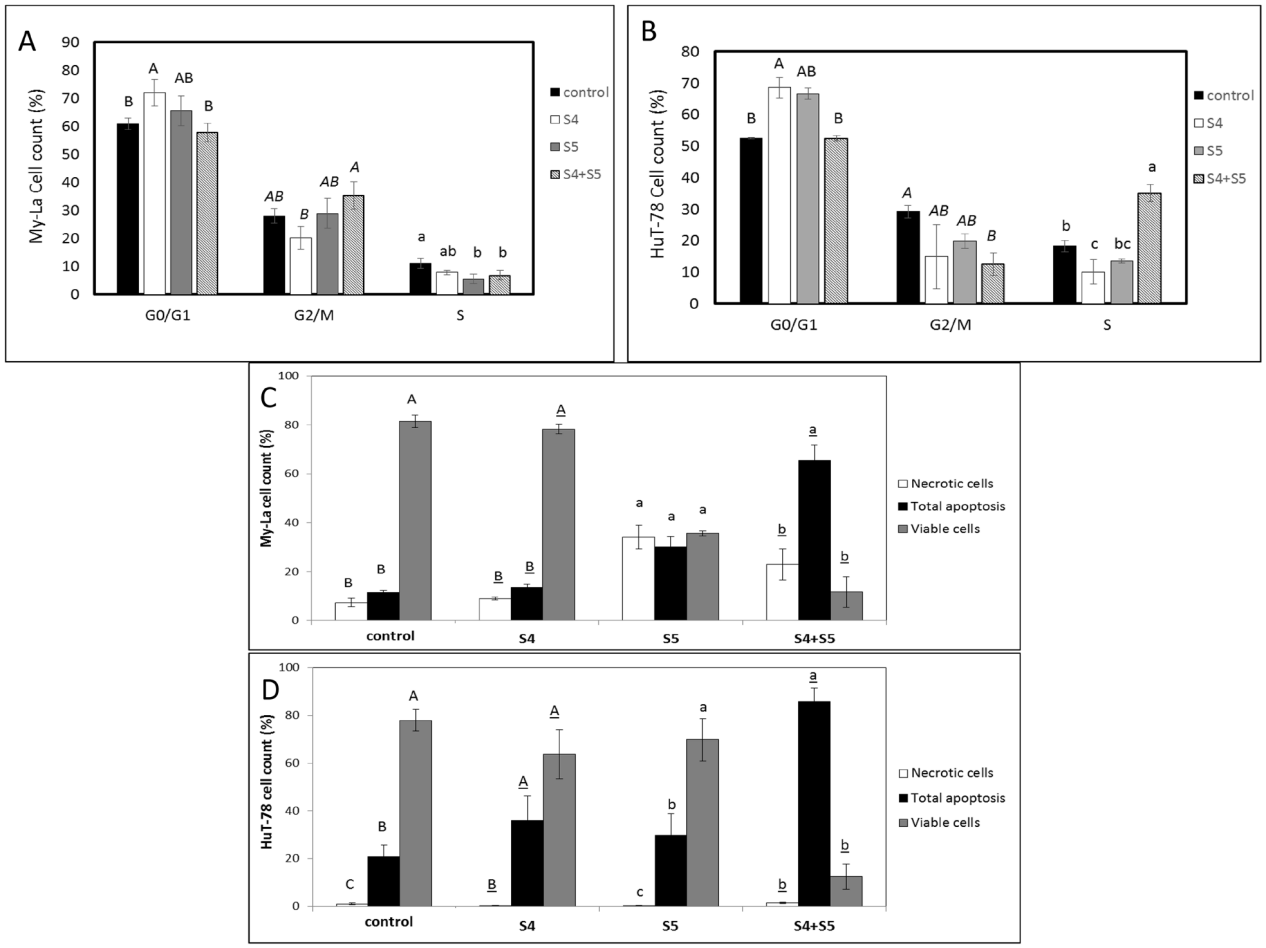
Determination of stages of cell cycle arrest following treatment with S4, S5, or S4+S5 on My-La (**A**) or HuT-78 (**B**) cell lines. Starved cells were treated with S4 (5 μg/mL), S5 (6 μg/mL), S4 (5 μg/mL) + S5 (6 μg/mL) and methanol (control) for 48 h. The treated cells were harvested, fixed, and analyzed in FACS following PI staining. The percentage of cells in G0/G1, G2/M and S phase were analyzed from 10,000 events per treatment. Error bars indicate ± SE (2 biological replicates were done, in each *n* = 3). Levels with different letters are significantly different from all combinations of pairs according to Tukey-Kramer honest significant difference (HSD; *P* ≤ 0.05). Determination of proportion of viable, apoptotic or necrotic cells following treatment with S4, S5, or S4+S5 on My-La (**C**) or HuT-78 (**D**) cell lines. Cells were treated with S4 (5 μg/mL), S5 (6 μg/mL), S4 (5 μg/mL) + S5 (6 μg/mL) and methanol (control) for 48 h. The treated cells were harvested and analyzed in FACS following Annexin V-FITC and PI staining. Shown are the percentages of viable, necrotic, and apoptotic cells, analyzed from 10,000 cells per treatment. FACS, fluorescence-activated cell sorting; PI, propidium iodide. Error bars indicate ± SE (2 biological replicates were done, in each *n* = 3). Levels with different letters are significantly different from all combinations of pairs according to Tukey-Kramer honest significant difference (HSD; *P* ≤ 0.05).

S4 (5 μg/mL) + S5 (6 μg/mL) treatment of My-La for 24 h led to slight enrichment in the percentage of cells in the G2-M phase of the cell cycle (35.3%), in comparison to 28.0% in the control (Figure 3A). The same S4+S5 treatment of HuT-78 led to 30% cells in the S phase in comparison to 18.3% in the control (Figure 3B).

Treatment of My-La with S4 (5 μg/mL) + S5 (6 μg/mL) for 48 h led to apoptosis in 65.4% of the cells in comparison to 11.3% in the controls (Figure 3C; Supplementary Figure 2A). The proportion of apoptotic cells in S5-only treated My-La cells at 48 h was lower (30.0%), although S5 treatment led to a high level of necrosis (34.0%). Treatment of My-La with S4 lead to slight increase in apoptotic cell death rates above the control treatment (19.8%; Figure 3C; Supplementary Figure 2A).

Total cell apoptosis for HuT-78 was 36.0% for S4 and 29.8% for S5 treatments alone (Figure 3D; Supplementary Figure 2B). For S4+S5 treatment total cell apoptosis for HuT-78 was 85.9%, in comparison to 20.9% for the control (Figure 3D; Supplementary Figure 2B).

### S4+S5 have cytotoxic and apoptotic activity on SPBL

In order to examine the therapeutic potential of S4+S5 treatment, we determined the activity of S4+S5 on SPBL cell viability. Effective concentrations on My-La and HuT-78 cell lines were also highly effective against SPBL. S4 (10 μg/mL) + S5 (20 μg/mL) treatment led to only 20.3% viable SPBL cells while lower concentration S4 (5 μg/mL) + S5 (6 μg/mL) treatment led to 56.0% viable SPBL cells (Table 1). Variable concentrations of S4 and S5 found to be only moderately cytotoxic to the cell lines (Table 1), were also only moderately active on SPBL (e. g., S4 [5 μg/mL] + S5 [2 μg/mL], 80.9% viable SPBL cells; Table 1).

**Figure 4:**
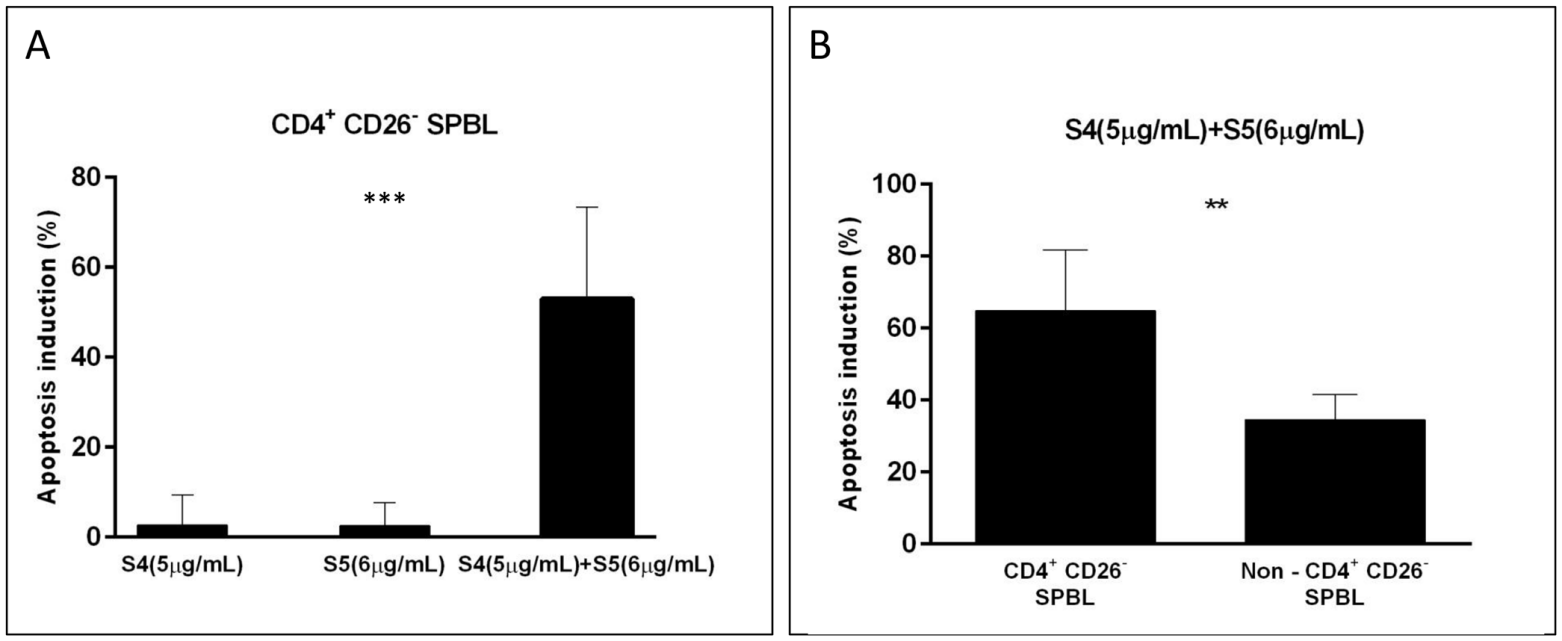
Apoptosis induction in PBL from Sézary patients following treatment with S4, S5, and S4+S5. PBL were isolated from blood samples of Sézary patients (*n* = 6), and were treated with S4 (5 μg/mL), S5 (6 μg/mL), S4 (5 μg/mL) +S5 (6 μg/mL) and control (methanol; Vehicle treatment) for 48 h. Cells were harvested and analyzed by FACS following CD4-APC, CD26-alexa 405, Annexin V-FITC and PI staining. Apoptotic-induced cells (Annexin positive cells) were determined in the CD4^+^CD26^-^ cell population and in non-CD4^+^CD26^-^ cells of treated cells minus control. (**A**) The percent of apoptotic-induced CD4^+^CD26^-^ cells is presented for single treatment compared to combined treatment; (*n* = 4); ^***^denote significant difference between means (one way ANOVA; *p* < 0.001). (**B**) The percent of apoptotic-induced cells following the combined treatment was compared between CD4^+^CD26^-^ cells and non-CD4^+^CD26^-^ cells of SPBL; (*n* = 6); ** denote significant difference between means (paired student *T* test; 0.001 *< P* < 0.05).

S4 and S5 were tested for their apoptotic effect on CD4^+^CD26^-^ cells of SPBL (*n* = 6) (CD4^+^CD26^-^ are considered markers for Sézary-enriched cell populations [20];). The average percentage of apoptosis induction by treatment with each fraction separately for the SPBL population was ~2.5% (*n* = 4), while treatment with a combination of S4 (6 μg/mL) + S5 (6 μg/mL) led to 53% apoptotic cells, suggesting a significant increase in apoptosis with the synergistic treatment (Figure 4A, Supplementary Figure 3A). The increase in apoptosis induction by the combined treatment compared to single treatment was significant (*p* = 0.0004). Moreover, the synergistic treatment led to a higher proportion of apoptotic cells in the malignant cell (CD4^+^CD26^-^) sub-population than in the non-malignant non-CD4^+^CD26^-^ sub-population of SPBL (*n* = 6). Apoptosis induction of SPBL (*n* = 6, with an average of 60.5% CD4^+^CD26^-^ cells) was 65%, whereas in the non-CD4^+^CD26^-^ counterpart, apoptosis induction was 31% (*p* = 0.0016) (Figure 4B, Supplementary Figure 3B). This suggests that the induction of apoptosis is selective to a significant extent for CD4^+^CD26^-^ cells.

### S4+S5 treatment leads to distinct gene expression profiles

**Figure 5:**
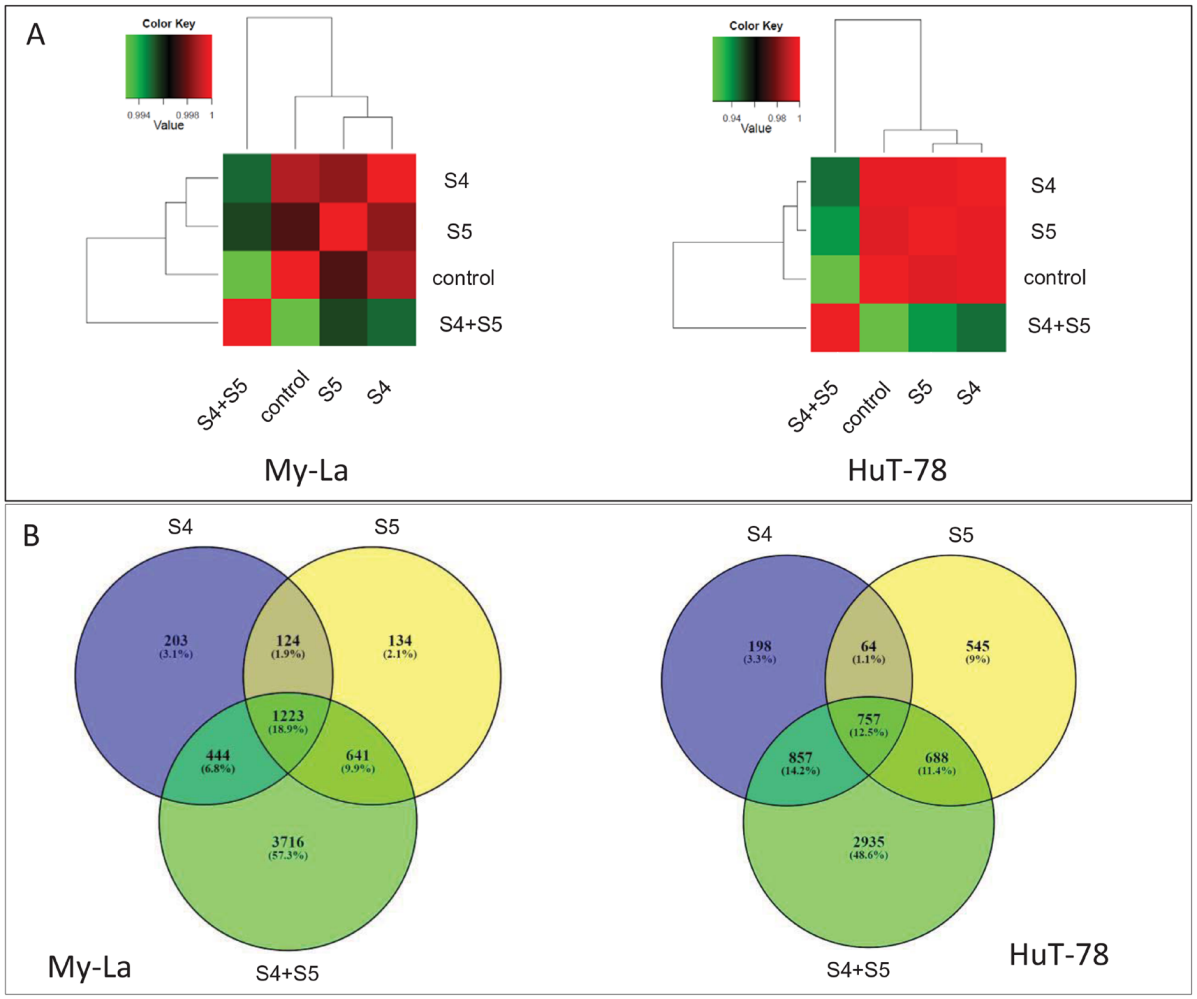
Hierarchical clustering and Venn diagram of genes significantly differentially expressed genes in My-La and HuT-78 cells treated with S4, S5 or the S4+S5 synergistic combination. (**A**) Hierarchical clustering using Pearson correlations among the four conditions based on the genes expression (average fragments per kilobase of transcript per million fragments mapped [FPKM] of the three replications) followed by a log2 transform. Pearson correlations were calculated with the R software package. (**B**) Venn diagrams illustrating the relationships between significantly differentially expressed genes (*padj* < 0.05) in the three treatments against the control. Venn diagrams were generated using the online tool at bioinformatics.psb.ugent.be/webtools/Venn/.

To identify genes and pathways differentially expressed in My-La and HuT-78 cell lines following treatment with SCBD fractions, we performed RNA sequence analysis of cells treated with S4 (5 μg/mL) or S5 (6 μg/mL) fractions separately, and with the S4+S5 synergistic combination. Sample correlation tests showed that RNA sequencing resulted in separate clusters for My-La versus HuT-78 cells (Figure 5A). In both cell lines, cells treated with S4 or S5 clustered together, and those of the control treatment clustered in a separate clade (Figure 5A). However, the cell lines treated with the synergistic S4+S5 combination clustered as an outgroup clade separate from the rest of the treatments (Figure 5A).

My-La or HuT-78 cells treated with the synergistic S4+S5 combination showed 3716 or 2935 genes, respectively, differentially expressed compared to the control and cells treated with S4 or S5 only (Figure 5B; *padj* < 0.05 ; DESeq2). Data on the differentially expressed genes are listed in Supplementary Table 3. Of these, 947 genes were differentially expressed in both My-La and HuT-78 cell lines following the synergistic S4+S5 treatment (compared to the control and to cells treated with S4 or S5 only) (Supplementary Table 4). Values of expression for genes differentially expressed in all treatments (S4, S5 and S4+S5) versus control are presented in Supplementary Table 5.

Accordingly, treatment with the S4+S5 synergistic combination led to the induction of several different biological pathways. The biological pathways with 10 or more genes that are significantly and at least 2-fold regulated in S4+S5 treatment versus control in both My-La and HuT-78 cell lines are listed in Supplementary Table 6. Involved pathways include, among others, the PHOSPHOINOSITIDE-3-KINASE–PROTEIN KINASE B (PI3K-AKT) pathway, as well as cancer and cytokine/chemokine-receptor interaction pathways (Supplementary Table 6).

The steady state RNA levels, determined by qPCR, of some of the genes differentially expressed after S4+S5 treatment (Supplementary Table 3) are presented in Table 2. In HuT-78 cells validated by qPCR were *NF-KAPPA-B INHIBITOR ZETA* (*NFKBIZ*; geneID 64332) and *special AT-RICH SEQUENCE-BINDING PROTEIN-1* (*SATB1*; geneID 6304) upregulation, and *PHOSPHOINOSITIDE-3-KINASE–PROTEIN KINASE B R3* (*PIK3R3*) (geneID 8503) repression by the S4+S5 treatment (Table 2). *RIBONUCLEOTIDE REDUCTASE REGULATORY SUBUNIT M2* (*RRM2*; geneID 6241; Supplementary Table 5) was significantly downregulated in My-La and HuT-78 cells. however it was shown by qPCR to be downregulated in HuT-78 cells only (Table 2). Transcription factors *ACTIVATING TRANSCRIPTION FACTOR 4* (*ATF4*; gene ID 468; Supplementary Table 5) and the *PSEUDOKINASE TRIBBLES HOMOLOGUE 3* (*TRIB3*; gene ID 57761; Supplementary Table 5) were significantly induced by the S4+S5 treatment in both the RNA sequencing data and in the qPCR experiments (Table 2). However the changes in expression of *AKT1* (geneID 207) and *KCNN4* (geneID 3783), both suggested by the RNA sequencing results to be repressed by S4+S5 treatment, could not be validated by qPCR.

**Table 2:** Quantitative PCR determination of the RNA steady state level in My-La and HuT-78 cell lines of genes found to be differentially expressed after treatment with S4 (5 μg/mL) + S5 (6 μg/mL) for 6 h relative to control

Cell line	Gene	RNA steady state level of S4+S5 treated versus control	Significantly changed in comparison to control (paired student *T* test; 0.001 < *P* < 0.05)
My-La	*NFKBIZ*	0.84 ± 0.01	+
HuT-78	*NFKBIZ*	3.93 ± 0.22	+
My-La	*RRM2*	1.07 ± 0.03	—
HuT-78	*RRM2*	0.66 ± 0.00	+
My-La	*SATB1*	1.02 ± 0.03	—
HuT-78	*SATB1*	3.95 ± 0.68	+
My-La	*PIK3R3*	1.41 ± 0.05	+
HuT-78	*PIK3R3*	0.58 ± 0.03	+
My-La	*AKT1*	1.43 ± 0.11	+
HuT-78	*AKT1*	1.63 ± 0.04	+
My-La	*KCNN4*	1.99 ± 0.14	+
HuT-78	*KCNN4*	1.20 ± 0.04	+
My-La	*ATF4*	1.47 ± 0.02	+
HuT-78	*ATF4*	3.00 ± 0.20	+
My-La	*TRIB3*	1.87 ± 0.07	+
HuT-78	*TRIB3*	14.95 ± 1.09	+

Gene transcript values were determined by quantitative PCR as a ratio between the target gene versus a reference gene (*HYPOXANTHINE PHOSPHORIBOSYLTRANSFERASE*1; *HPRT1*; geneID 3251). Values for My-La or HuT-78 cells treated with S4 (5 μg/mL) + S5 (6 μg/mL) were calculated relative to the average expression of target genes in My-La or HuT-78 methanol-treated controls using the 2^∆∆Ct^method. + indicates significance.

Examination of steady state RNA levels of some of the differentially expressed genes in SPBL following S4 (5 μg/mL) + S5 (6 μg/mL) treatment versus the control revealed that *SATB1* (geneID 6304) was upregulated in all examined SPBL (Table 3). However, only slight changes in gene expression versus the control were recorded for *NFKBIZ* (geneID 64332), whereas *RRM2* (geneID 6241) expression was slightly repressed in 2 out of 3 examined patients (Table 3). *PIK3R3* (geneID 8503) expression was slightly increased in SPBL after S4+S5 treatment (Table 3).

**Table 3:** The RNA steady state level in Sézary patients peripheral blood lymphocytes (SPBL) of genes differentially expressed in CTCL cell lines treated with S4 (5 μg/mL) + S5 (6 μg/mL) for 48 h relative to the control

Patient designation/target gene	*SATB1* Gene ID 6304	*NFKBIZ-1* Gene ID 64332	*RRM2-9* Gene ID 50484	*PIK3R3-1* Gene ID 8503
Sz-13	1.23 ± 0.10	0.96 ± 0.14	0.78 ± 0.07	1.03 ± 0.09
Sz-14	5.20 ± 0.38	0.90 ± 0.15	0.72 ± 0.02	1.19 ± 0.01
Sz-15	1.20 ± 0.32	1.04 ± 0.09	1.07 ± 0.03	1.45 ± 0.26

Gene transcript values were determined by quantitative PCR as a ratio between target genes versus a reference gene (*hypoxanthine phosphoribosyltransferase*, *HPRT*, geneID 3251). Values for SPBLs treated with S4 [5 μg/mL] + S5 [6 μg/mL] were calculated relative to the average expression of target genes in the SPBL methanol-treated control, using the 2^∆∆Ct^ method.

## DISCUSSION

We have shown that a certain synergistic mixture of phytocannabinoids derived from *C. sativa* extracts have significant cytotoxic activity against My-La and HuT-78 cell lines and against SPBL. Ethanol extracts of a high-CBD cannabis strain exhibited cytotoxic activity against My-La cells. Fractionation of the whole extract led to the identification of fractions that exhibited significantly higher cytotoxic activity than the whole extract. Similar cytotoxic activity was found for the fractions also against the HuT-78 cell line. Moreover, the two active fractions, S4 and S5, were found to act synergistically on My-La and significantly on HuT-78 cell line under certain concentrations. These results suggest that fractionation may allow the selection of active compounds and depletion of non-active or antagonistic components present in the whole extract, leading to higher specific activity. Further, the synergistic interaction between the active fractions (S4 and S5) increased cytotoxicity and reduced the concentrations of these fractions needed for significant activity.

S4 contained CBD and minute proportions of THC, CBG, a-bisabolol and CBDV whereas S5 contained primarily CBD and CBG, as well as THC and CBC in low concentrations. Pure CBD applied at the concentration found in the S4+S5 treatment was significantly not or less cytotoxic to the cell lines than S4+S5 in My-La and HuT-78 cell lines, respectively. Also, in both cell lines, only treatment with a combination of the phytocannabinoids that compose S4 and S5 led to cytotoxicity similar to that of S4+S5 treatment. Hence, we suggest that the combined treatment by S4+S5 is not merely an additive effect of CBD (present in both fractions) but is based on genuine interaction between the different phytocannabinoids present in S4 and S5. In line with our findings, previous research showed that combinations of CBD and CBG exhibit strong synergistic cytotoxic activity against leukemia cell lines [17]. Taken together with earlier observations, synergy may have implications regarding dosage for therapy, including possible reduction of dosage when using synergistic cannabinoid combinations without a significant loss of activity.

Treatment with the synergistic combination of the active fractions led to apoptotic cell death in My-La and HuT-78 cell lines. Moreover, the synergistic treatment also led to apoptosis in SPBL, which was significantly selective to the malignant enriched cell population within the SPBL, further implicating possible therapeutic use. Indeed, a prevalent effect of cannabinoids on cancer cells is the induction of death by apoptosis and the inhibition of cancer cell proliferation [21]. For example, THC was previously demonstrated to induce the apoptotic death of glioma cells via CB_1_ and CB_2_ receptors. This activity was shown to induce expression of the endoplasmic reticulum (er) stress-related transcription factors *ATF4* (gene ID 468) and *TRIB3* (gene ID 57761) [22]. In our dataset, *ATF4* and *TRIB3* were significantly upregulated with treatment of S4, S5 and even more significantly with the S4+S5 combination. Induction by S4+S5 treatment was validated by qPCR. Hence, although the involvement of CB receptors was not demonstrated here and additional studies should be conducted, induction of stress-related genes by the S4+S5 treatment may be suggested, implying induction of er-stress similarly to that demonstrated for THC [21, 22].

Non-Hodgkin lymphoma cells expressed higher mRNA levels of CB_1_ and/or CB_2_ receptors compared to reactive lymphoid tissue [23], and mantle cell lymphoma consistently overexpressed CB_1_ and CB_2_ in comparison to normal purified B lymphocytes and reactive lymphoid tissue [24]. Among phytocannabinoids, only THC has been previously shown to bind and activate CB receptors [6]. CBD was previously found to be a non-competitive negative allosteric modulator of CB_1_ [25]. However, we could not detect consistent differences in CB_1_ and CB_2_ gene expression between HPBL and SPBL. Additionally, CB gene expression data did not correspond to the interference of CBs inverse agonists with activity in our study. This may imply that S4+S5 activity is not simply mediated via CB_1_ or CB_2_ but perhaps through other receptors or non-receptor pathways.

We found synergistic activity for the S4+S5 combinations and for the corresponding mixture of CBD, CBG, THC and CBC in My-La and HuT-78 cell lines. This synergy is dependent on the specific ratios between these compounds. The synergy in S4+S5 activity might result from the enhanced activation by the mixture of phytocannabinoids in comparison to a single phytocannabinoid of other receptors besides CB_1_ or CB_2_, or through other non-receptor dependent pathways. However, this requires further study.

Synergy may also result from the activation of more than one signaling pathway [26]. For further insight into the effect of S4+S5 on CTCL, we profiled the gene expression of My-La and HuT-78 cells treated with S4, S5, or with the synergistic S4+S5 combination. The synergistic treatment led to differential gene expression when compared to the control. This gene expression profile was substantially different from those obtained for S4 or S5 treatments, suggesting that the synergistic treatment induces different molecular events.

Several genes and corresponding proteins previously described in MF were found to be significantly affected in My-La cells by S4+S5 treatment. Of these, *NFKBIZ* (geneID 64332), a gene encoding NF-kB signaling inhibitor, related to the cytokine-cytokine receptor interaction pathway, was upregulated by S4+S5 treatment in My-La and HuT-78 cells but not in SPBL. *NFKBIZ* upregulation was validated by qPCR in HuT-78 cells. This upregulation suggests a possible reduction of NF-kB activity (a hallmark in MF [27];).

*RRM2* (geneID 6241) was downregulated in My-La cells under S4+S5 treatment; however qPCR results demonstrated reduction in *RRM2* expression in HuT-78 cells. *RRM2* is upregulated in tumor stage MF and its downregulation by S4+S5 is an interesting observation as *RRM2* may be a relevant target for S4+S5 treatment. This is because when *RRM2* is upregulated it induces NF-kB-dependent MMP9 activation, enhancing cellular invasiveness [28]. Several potent inhibitors for *RRM2* protein have been described that suppress tumor growth [29]. *RRM2* was downregulated by the S4+S5 treatment in 2 out of 3 examined Sézary patients SPBL.

In HuT-78 cell lines S4+S5 treatment induced expression of *SATB1* (geneID 6304). Importantly, *SATB1* induced expression by S4+S5 treatment was verified by qPCR in HuT-78 cells and was found to also be induced in SPBL of the examined patients. Deficient *SATB1* expression hampers T-cell development and results in misregulation of T-cell lineages [30, 31]. Previous studies suggested that dysregulated *SATB1* expression is involved in CTCL progression: low *SATB1* was associated with an impaired prognosis, although *SATB1* expression did not correlate with MF stages in all studies [32]. Recent data provided mechanistic evidence that the proto-oncogenic JAK3-STAT5 pathway promotes an aberrant expression of IL-5 and IL-9 through miR-155-mediated repression of *SATB1* [33].

However, the expression of 2 other genes was reduced in HuT-78 cells under S4+S5 treatment, with potentially unfavorable outcomes. One was *STAT4* (geneID 6775; *SIGNAL TRANSDUCER AND ACTIVATOR OF TRANSCRIPTION* 4) required for the development of Th1 cells from naïve CD4^+^ T cells and IFN-γ production in response to IL-12 [34, 35]. The second was *BCL2L11* (geneID 10018), an apoptotic activator whose loss is also involved with CTCL [36].

As mentioned above, the PI3K-Akt signaling pathway was affected in both My-La and HuT-78 cells by the synergistic S4+S5 treatment. The PI3K pathway is pivotal in normal and malignant lymphocyte biology [37] and class 1A PI3Ks have been associated with many human cancers as oncogenic drivers [38]. Reducing PI3KR3 activity is considered an attractive target in anticancer therapy for multiple tumor suppression including lymphomas, and agents targeting these components have been developed [37]. *PI3K* expression is frequently constitutively active in lymphomas as a result of gene amplification or duplication of wild type *PIK3CA*, which encodes a subunit of PI3K, wherein inhibition of PIK3CA activity results in cell apoptosis [37]. qPCR experiment suggested significant repression of *PIK3R3-1* in HuT-78 and an increase of its expression in My-La cells following S4+S5 treatment. However, in SPBL *PIK3R3* expression was slightly increased.

Expression of several cyclins and cyclin-dependent kinases associated with cell cycle progression was altered by the S4+S5 treatment based on the RNA sequencing data (e. g., *CCND2*, *CCND3*, *CCNE1, CCNE2, CDK19; CNL2*; Supplementary Table 3); cell cycle associated proteins may be potent inhibitors of cell proliferation in cancer cells leading to cell apoptosis [39], and therefore may be a desired therapeutic target. Cell sorting experiments in our studies show that S4+S5 treatment led to slight G2-M arrest in the My-La cell line and to a significant S phase arrest in the HuT-78 cell line, in addition to cell apoptosis in both My-La and HuT-78 cell lines.

To conclude, active cannabis extract fractions and their synergistic combinations were cytotoxic to CTCL cell lines in *in-vitro* and to SPBL in *ex-vivo* studies. The defined S4+S5 formulation of synergistic phytocannabinoids induced cell cycle arrest and cell apoptosis, and affected multiple biological pathways, including those associated with cancer. Based on this pre-clinical study new cannabis-based products that are based on precise composition of synergistically interacting compounds may be developed. Human clinical trials are needed to validate the effectiveness of these synergistic cannabis compounds of active pharmaceutical ingredients for the treatment of CTCL.

## MATERIALS AND METHODS

### Extraction of *cannabis sativa* inflorescence

Fresh or dry specimens of *C. sativa* inflorescence strains DQ and SCBD were obtained from IMC. After extraction they were immediately frozen at –20°C using liquid nitrogen. Frozen fresh/dry inflorescences were ground by mortar and pestle and placed in 15 mL tubes. Absolute ethanol was added to each inflorescence powder sample at a sample-to-absolute ethanol ratio of 1:4 (w/v). The samples were mixed thoroughly on a shaker for 30 min, and then the extract was syringe filtered (0.2 PVDF syringe filter). The filtrate was transferred to new tubes. The solvent was evaporated under nitrogen. The dried extract was weighed, and then resuspended in absolute methanol (volume of solvent added according to the desired concentration) and filtered through a 0.45 μm syringe filter. For the treatments, the resuspended extract was diluted according to cell cultures.

### Chemical characterization

#### Standard preparation

The phytocannabinoid standards cannabigerol (CBG, Restek catalog no. 34091), cannabidiol (CBD, Restek catalog no. 34011), ∆-9 tetrahydrocannabinol (∆-9 THC, Restek catalog no. 34067) and cannabichromene (CBC, Restek catalog no. 34092) were received in concentration of 1 mg/mL, originally dissolved in methanol. Standards were diluted in medium in ratio of 1:10 for XTT assays (described below). For quantification of phytocannabinoids by analytical HPLC (described below), the standards were dissolved in methanol at different concentrations from 5 ppm to 60 ppm.

#### HPLC analysis and sample separation

For analytical HPLC, the dry crude extract was resuspended in methanol (MeOH) and filtered through a 0.45 μm syringe filter (Merck, Darmstadt, Germany) and 20 μL of the filtered extract was injected. Sample profiles were obtained from an UltiMate 3000 HPLC system coupled with WPS-3000(T) Autosampler, HPG-3400 pump, and DAD-300 detector. The separation was performed on a Raptor ARC-18 column, 2.7 μm, 150 × 4.6 mm (Restek, 9314A65). Diluent: 25:75 water: methanol, Inj. Vol: 5 μL. Mobile phase: A: water, 5 mM ammonium formate, 0.1% formic acid; B: acetonitrile, 0.1% formic acid. Isocratic (%B): 0 min (75%), 9 min (75%) at a flow rate of 1.5 mL min^-1^. The compound peaks were detected at 220 and 280 nm.

For preparative HPLC 50 mg of the dry crude extract was dissolved in 10 mL solvent (75% MeOH and 25% water containing 0.1% acetic acid), and filtered through 0.45 μm syringe filter. Then, 10 mL of the filtered extract were injected. Sample separation in preparative HPLC was carried out using a Agilent Technologies 1260 Infinity preparative HPLC system, 1260 MWD-VL detector, Column: Kinetex 5u EVO C18 100A, 250 × 21.2 mm (Phenomenex). Mobile phase: A: water, 0.1% acetic acid, B: methanol. Gradient: (%B): 0.00 min (60%), 45 min (85%) for total run of 55 min.

### Gas chromatograph (GC) with mass selective detector (MSD) (GC/MS) analysis

GC/MS analyses were carried out following [19], using a HP7890 gas chromatograph coupled to a HP6973 mass spectrometer with electron multiplier potential 2 KV, filament current 0.35 mA, electron energy 70 eV, and the spectra were recorded over 40 to 400 m/z. For concentration, 1 mg of each sample was dried under a gentle stream of nitrogen, and then dissolved in 6 mL of methanol prior to introduction to GC/MS.

### Cell cultures

My-La and HuT-78 cells were generously donated by Robert Gniadecki, MD (Copenhagen University, Copenhagen, Denmark). My-La CD4+ cell line (95051032, ECACC) was established from skin biopsy of 82-year-old Caucasian male with 80% Body Surface Area (BSA) involvement by MF with extensive lymphadenopathy (stage IIA) [40]. HuT-78 cell line (TIB-161, ATCC) was established from PBL of 53-year-old Caucasian male with Sézary involving skin, blood, lymph nodes and liver [41]. Cells were grown at 37°C in a humidified 5% CO_2_–95% air atmosphere. Cells were maintained in Dulbecco’s Modified Eagle Medium (DMEM, for My-La cells) and RPMI1640 medium (for HuT-78 cells).

### Isolation of human peripheral blood lymphocytes

Peripheral blood was diluted 1:2 in sterile phosphate-buffered saline (PBS). Same volume of Lymphoprep (STEMCELL Technologies) was added to the peripheral blood sample with a Pasteur pipette, and the sample was centrifuged (800 × g, 20 min, 20°C). PBL were collected from the white median interphase, rinsed twice with PBS, and suspended in RPMI medium with 10% Fetal Bovine Serum (FBS) to 2 × 10^6^ cells/mL.

### XTT cell viability assays

Cells in normal growing media were seeded into a 96-well plates at a concentration of 10,000 cells per well in triplicate. The following day, cells were treated with different extracts/fractions/compounds, or media/solvents alone for controls. In experiments where CB_1_ antagonist AM251 (Tocris, 1117/1) or CB_2_ inverse agonist SR144528 (Abcam, ab146185) were used, cells were treated with the antagonist/inverse agonist 1 h prior to treatments. Viability was quantified using the XTT viability assay (Biological Industries). Cells were incubated in medium for 48 h, then XTT reagent (2,3,-bis (2-methoxy- 4-nitro- 5-sulfophenyl)- 5-[(phenylamino)- carbonyl]-2H- tetrazolium inner salt) was added for 2 h at 37°C in a humidified 5% CO_2_–95% air atmosphere. Absorbance was recorded by a Synergy H1 hybrid reader photometer (BioTek) at 490 nm with 650 nm of reference wavelength. Cell survival (% viability) was estimated using the equation: % cell survival= (A_490_-A_650_) of treatment / (A_490_-A_650_) of solvent control × 100. A_490_ and A_650_ are the absorbencies of the XTT colorimetric reaction. Absorbance of medium alone (blank) was also reduced from specific readings.

### Analysis of combined drug effects

In order to examine if there was synergy between SCBD fractions S4 and S5, XTT assay was used to measure the toxicity of these fractions on My-La cells. Cells were seeded and treated as previously described in various concentrations. Drug synergy was determined by the Bliss independence drug interaction model which is defined by the following equation: Exy = Ex + Ey – (ExEy), where (Exy) is the additive effect of the drugs x and y as predicted by their individual effects (Ex and Ey) [42].

### Apoptosis assay for CTCL cell lines

Apoptosis for CTCL cell lines was assessed using MEBCYTO Apoptosis Kit with Annexin V-FITC and PI (MBL, Enco, 4700). Staining was according to manufacturer instructions. In brief, cells were seeded in 6-well plate culture dishes, at density of 1 × 10^6^ cells per well in DMEM (for My-La cells) or in RPMI (for HuT-78 cells). The following day, the media was replaced with new media containing S4 at a concentration of 5 μg/mL, S5 at a concentration of 6 μg/mL, a combination of S4 and S5, and methanol as control. Cells were then incubated for 48 h at 37°C in a humidified 5% CO_2_–95% air atmosphere. After incubation, cells were harvested and collected separately. Tubes were then centrifuged for 10 min at 900 g and cell pellets were resuspended and washed twice with 1 mL of PBS. The cells in each sample were resuspended in 85 μL of Annexin binding buffer. Cells were stained using 10 μL of Annexin V- FITC solution and 5 μL of propidium iodide (PI) working solution followed by incubation in darkness at room temperature for 15 min. Then 400 μL of Annexin V binding buffer were added to each tube and flow cytometry was performed using a Gallios flow cytometer (FACS). Cells were considered to be apoptotic if they were Annexin V+/PI- (early apoptotic) or Annexin V+/PI+ (late apoptotic). Live cells were defined as Annexin V-/PI-, and Annexin V-/PI+ as necrosis.

### Cell cycle analysis for CTCL cell lines

Cells were seeded in 6-well plate culture dishes at a concentration of 2 × 10^5^ cells/mL, 10,000 cells per well. After 24 h of incubation, cells were treated with S4 (5 μg/mL), S5 (6 μg/mL), a combination of S4 and S5, or methanol as control for another 48 h. Cells from each well were then harvested and collected separately and centrifuged for 10 min at 900 g. The cell pellet was washed once with 1 mL of PBS and fixed with 70% cold ethanol at 4°C for 1 h. The fixed cells then were pelleted out and washed twice with 1 mL of PBS. The cell pellet was then stained by resuspending in 250 μL of PI solution (50 μg/mL) containing RNase A (100 μg/mL) for 15 min in darkness. Then 400 μL of PBS were added to each tube and the cells were analyzed using FACS.

### PBL donors

Samples were collected from 7 Sézary patients at the Department of Dermatology, Rabin Medical Center, Petah Tikva, Israel. All had Sézary according to the revised staging criteria [43], with clinical stage IVA disease. In addition, blood samples enriched with PBL were obtained from leftover blood of 4 healthy blood donors at Magen David Adom, Sheba Medical Center, Israel. All patients provided their written informed consent to participate in the study, approved by the Ethics Committee of Rabin Medical Center (Ref. 6515 for PBL from healthy subjects and Ref. 7175 for PBL from patients with SS).

### Apoptosis assay for SPBL

SPBL (1 × 10^6^ cells) were washed with PBS and then with binding buffer (BMS-500FI, eBioscience). Cells were suspended in 100 μL binding buffer with 1 μL Annexin V-FITC (4830-01-1, eBioscience) + 2 μL CD26 Alexa Fluor 405-conjugated antibody (FAB1180V-100UG, R&D SYSTEMS) + 10 μL CD4-APC-conjugated antibody (FAB3791A-100, R&D SYSTEMS) and incubated for 15 min at room temperature. Cells were washed with binding buffer and suspended in 190 μL binding buffer + 1 μl of PI (00-6990-42, eBioscience). 300 μL of PBS were added and samples were analyzed by FACS. The percent of apoptotic cells (annexin positive cells) was determined in CD4^+^CD26^-^ gated lymphocytes and in non-CD4^+^CD26^-^ gated lymphocytes. The apoptosis induction of each treatment was obtained by reducing the percent of apoptotic cells treated with the methanol control from the percent of apoptotic cells treated with the fractions.

### Quantitative real-time (qRT) PCR

Cells were seeded into a 12-well plate at a concentration of 2 × 10^6^ cells/mL. After 24 h incubation at 37°C in a humidified 5% CO_2_–95% air atmosphere, cells were treated with combination of S4 and S5 (at 5 μg/mL and 6 μg/mL respectively) and methanol as a control for 48 h. Cells were then harvested and total RNA was extracted using TRI reagent (Sigma-Aldrich) according to the manufacturer’s protocol. For cell lines all treatments were added for 6 h, for PBL all treatments were added for 48 h. RNA was reverse-transcribed in a total volume of 20 μL using Maxima reverse transcriptase (Thermo Scientific) according to manufacturer’s protocol. All primers were designed using Primer3Plus software. PCR was performed in triplicate using a StepOnePlus system (Applied Biosystems). The expression of each target gene was normalized to the expression of HPRT mRNA in the 2^-∆∆Ct^ and is presented as the ratio of the target gene to HPRT mRNA, expressed as 2^-∆Ct^, where Ct is the threshold cycle and ∆Ct = Ct Target - Ct HPRT. Experiments were repeated three times. The primers were: *AKT1* (forward) 5′-GCTCACCCAGTGACAACTCA-3′ and (reverse) 5′- CCCAGCAGCTTCAGGTACTC-3′; for *SATB1* (forward) 5′-TGGTAAACCTTCGGGCTATG-3′ and (reverse) 5′-CCATTCCTTTCAGTGGCAAT-3′; for *RRM2* (forward) 5′-CCTCAGGTGACCTCTCCAAG-3′ and (reverse) 5′-TACTATGCCATCGCTTGCTG-3′; for *NFKBIZ* (forward) 5′-GGCAGCTGAAGAAGCAAATC-3′ and (reverse) 5′-TCAACCGATACTGCAAGCTG-3′; for *KCNN4* (forward) 5′-CATCACATTCCTGACCATCG–3′ and (reverse) 5′-ACGTGCTTCTCTGCCTTGTT-3′; for *PIK3R3* (forward) 5′-AGCCTGTGGAAATGGCATAG-3′ and (reverse) 5′-CTCTCATGAAGGAGGCCAAG-3′; for *ATF4* (forward) 5′-GGAAACCATGCCAGATGACC-3′ and (reverse) 5′-ACTTTCTGGGAGATGGCCAA-3′; for *TRIB3* (forward) 5′-GGTGCTTATCAGGTGCCAAG-3′ and (reverse) 5′-GTTGTCAGCTCAAGGATGCC-3′.

### RNA sequencing and transcriptome analysis

For RNA preparation, cells were seeded into a 6-well plate at a concentration of 1.5 × 10^6^ cells/mL per well. After 24 h of incubation at 37°C in a humidified 5% CO_2_–95% air atmosphere, cells were treated with S4 (5 μg/mL), S5 (6 μg/mL), a combination of S4 and S5 at these concentrations, and methanol for 6 hrs. The cells were subsequently harvested and total RNA was extracted using a TRI reagent (Sigma-Aldrich) according to manufacturer’s protocol. The RNA was kept at –80°C until further analysis. Sequencing libraries were prepared using the INCPM mRNA Seq protocol. Sixty bp single reads were sequenced on one lanes of an Illumina HiSeq. Transcriptome analysis was carried out as described in [19]. Briefly, the raw-reads were subjected to a filtering and cleaning procedure and FASTX Toolkit (http://hannonlab.cshl.edu/fastx_toolkit/index.html, version 0.0.13.2) was used to trim read-end nucleotides with quality scores < 30, and to remove reads with less than 70% base pairs with a quality score ≤ 30, using the FASTQ Quality Filter. Clean-reads were aligned to the human genome extracted from National Center for Biotechnology Information (NCBI) (GRCh38; https://www.ncbi.nlm.nih.gov/genome/guide/human/) using Tophat2 software (v2.1). Gene abundance estimation was performed using Cufflinks (v2.2) combined with gene annotations from the NCBI. Heatmap visualization was performed using R Bioconductor and differential expression analysis was completed using the DESeq2 R package. Genes that varied from the control more than twofold, with an adjusted *P*-value of no more than 0.05, were considered differentially expressed. The KEGG database (http://www.genome.jp/kegg/) was used for pathway analysis using the KEGG mapper tool (http://www.genome.jp/kegg/tool/map_pathway2.html). Enrichr tool was used for pathway enrichment analysis (http://amp.pharm.mssm.edu/Enrichr/).

## SUPPLEMENTARY MATERIALS












