# Supplementary material for: Synergistic cytotoxic activity of cannabinoids from cannabis sativa against cutaneous T-cell lymphoma (CTCL) in-vitro and ex-vivo
Source: Oncotarget. 2020 Mar 31;11(13):1141–56. doi: 10.18632/oncotarget.27528 (PMC7138167; doi:10.18632/oncotarget.27528)
Supplement: Supplementary file 1 [file oncotarget-11-1141-s001.pdf]

Synergistic cytotoxic activity of cannabinoids from *cannabis sativa* against cutaneous T-cell lymphoma (CTCL) *in vitro* and *ex vivo*

SUPPLEMENTARY MATERIALS

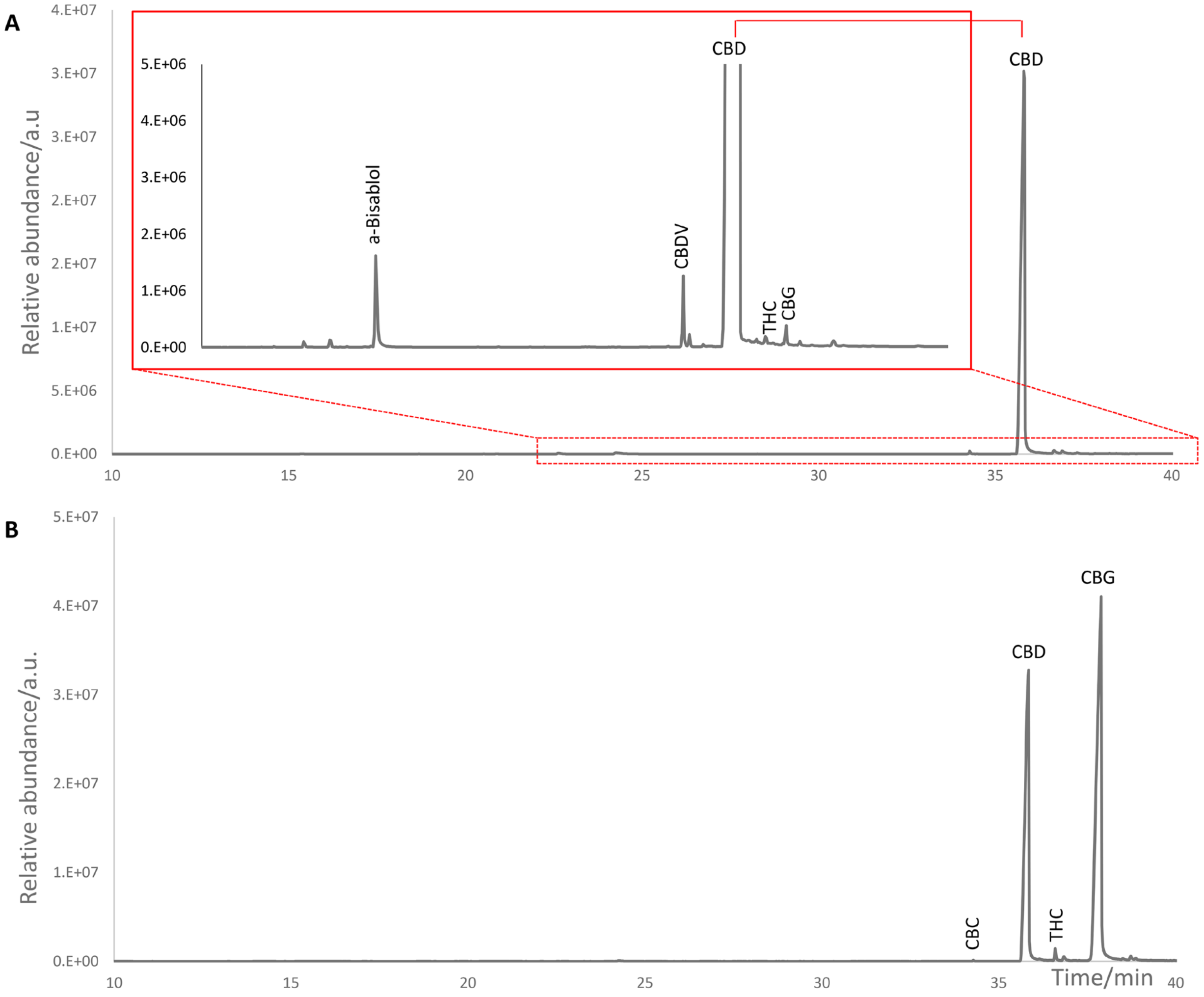

Supplementary Figure 1: GC-MS chromatograms of (**A**) S4 and (**B**) S5 fractions. Peaks are annotated for the terpene alcohols and decarboxylated cannabinoids present in the ethanolic extract, as detected in the GC. Relative amounts of the compounds detected can be extrapolated from the area under the peak. Minute peaks detected in (A) are enlarged as an insert.

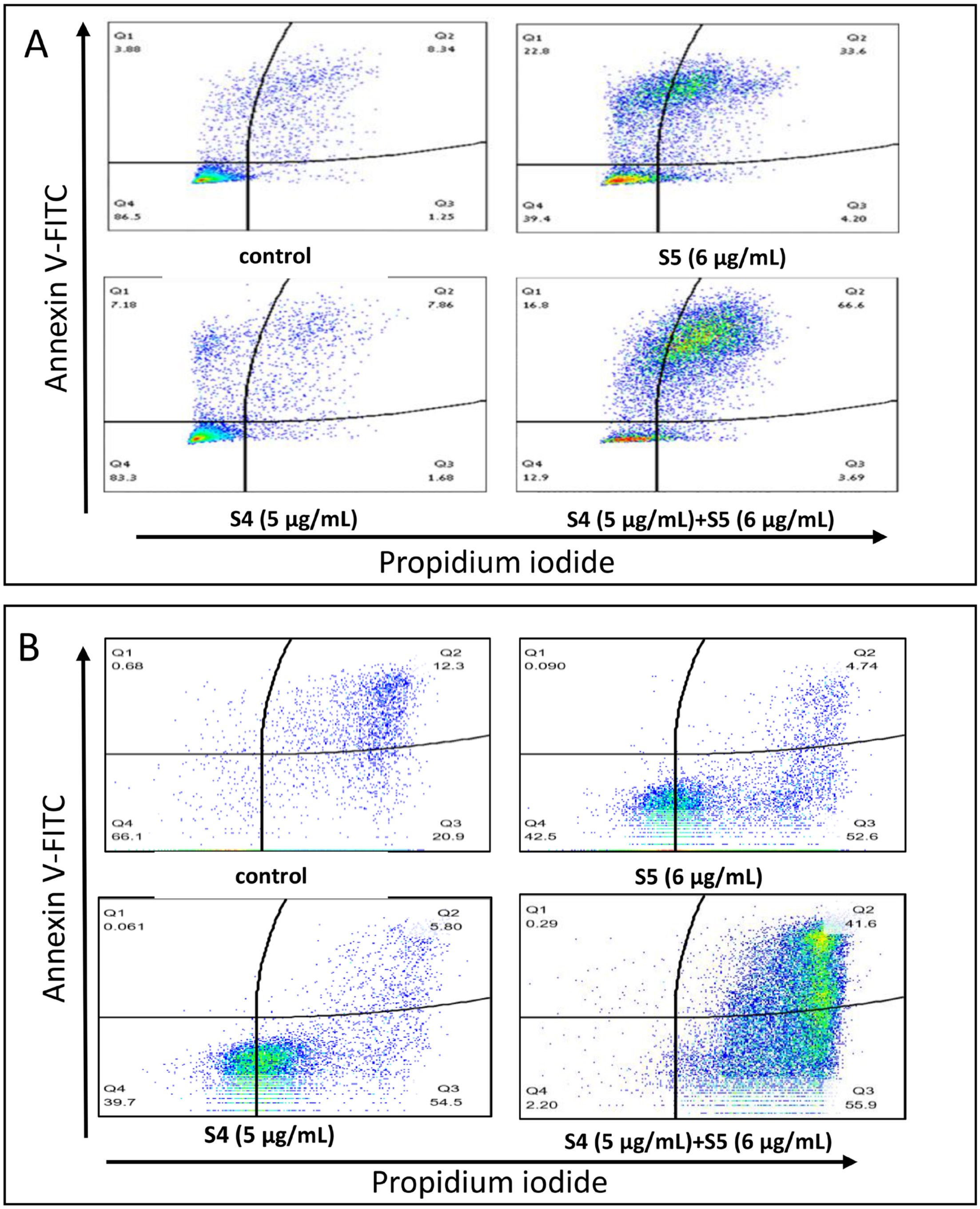

Supplementary Figure 2: Output of FACS following Annexin V-FITC and PI staining for determination of proportion of viable, apoptotic or necrotic cells following treatment with S4, S5, or S4+S5 on My-La (**A**) or HuT-78 (**B**) cell lines. Cells were treated with S4 (5 µg/mL), S5 (6 µg/mL), S4 (5 µg/mL) + S5 (6 µg/mL) or control (methanol) for 48 h. The treated cells were harvested and analyzed in FACS following Annexin V-FITC and PI staining. The histogram for each sample was split into four quadrants to indicate viable cells (lower left quadrant, Q4), early apoptotic cells (lower right quadrant, Q3), necrotic cells (upper left quadrant, Q1), and late apoptotic cells (upper right quadrant, Q2).

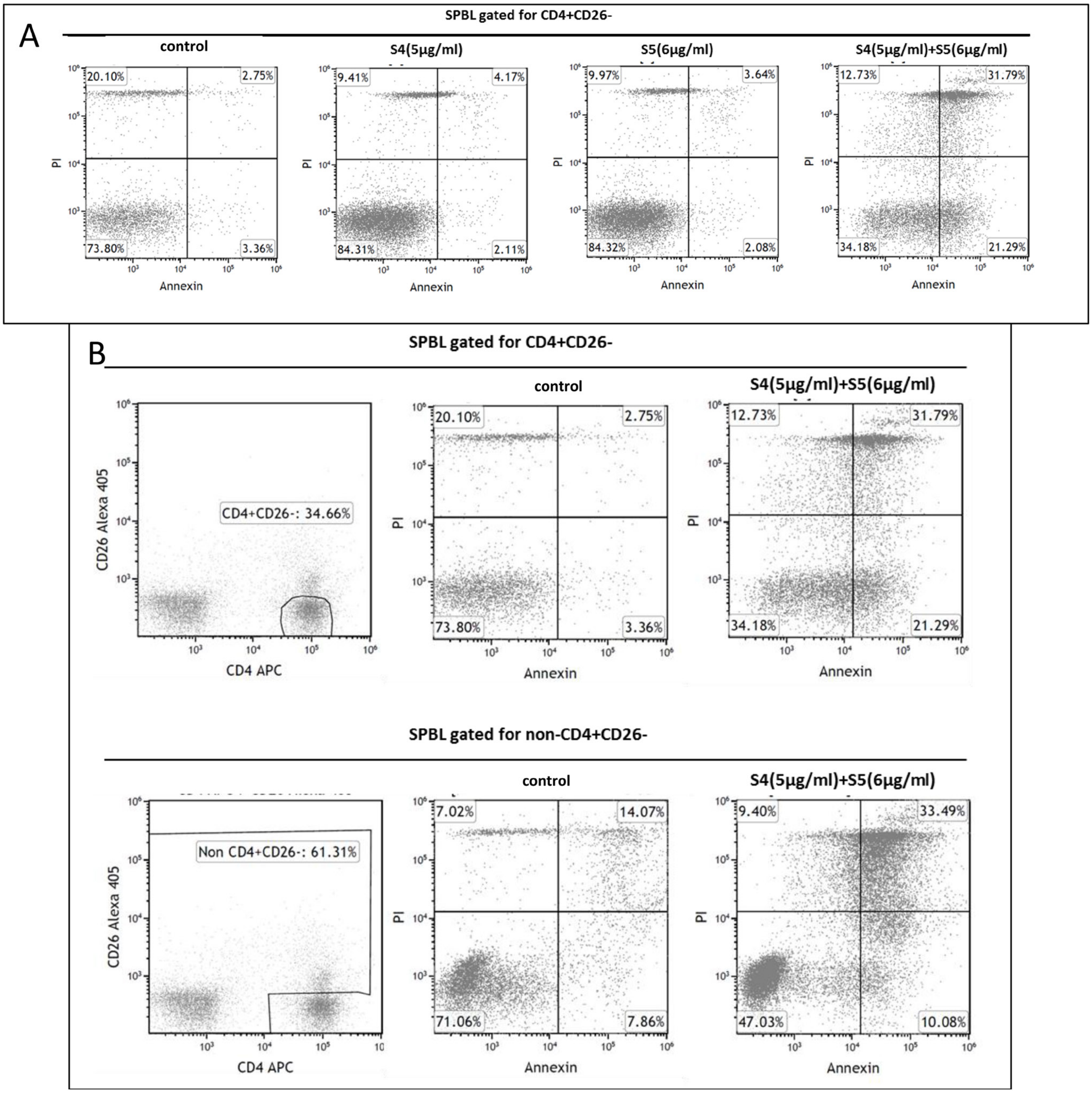

Supplementary Figure 3: Representative scatter plot of FACS analysis for the apoptosis induction of PBL from Sézary patients following treatment with S4 (5 µg/mL), S5 (6 µg/mL), S4 (5 µg/mL) + S5 (6 µg/mL) subjected to Figure 4. Cells treated with methanol (drug vehicle) were considered as control. (**A**) The percent of apoptotic-induced $CD4^{+}CD26^{-}$ cells is presented for single treatment compared to combined treatment. (**B**) The percent of apoptotic-induced cells following the combined treatment was compared between $CD4^{+}CD26^{-}$ cells and non-$CD4^{+}CD26^{-}$ cells of SPBL.

Supplementary Table 1: RNA levels of CB1 and CB2 in control and following treatment with S4 (5 µg/mL) + S5 (6 µg/mL). See Supplementary Table 1

Supplementary Table 2: Basal gene expression levels of CB_1 and CB_2 receptors in peripheral blood lymphocytes of Sézary patients (SPBL) relative to those of healthy individuals (HPBL)

SPBL	CB_1 relative gene expression level	CB_2 relative gene expression level
Sz-13	5.07 ± 0.53	2.73 ± 0.25
Sz-14	1.15 ± 0.02	0.77 ± 0.09
Sz-15	0.90 ± 0.14	0.79 ± 0.02
Sz-4	1.17 ± 0.08	0.54 ± 0.03

Values of gene transcripts were determined by quantitative PCR as a ratio between target genes (CB_1 and CB_2) versus a reference gene (*hypoxanthine phosphoribosyltransferase*, *HPRT*, geneID 3251). Values for SPBLs were calculated relative to the average expression of CB_1 and CB_2 receptor genes in peripheral blood lymphocytes of healthy individuals (HPBL, *n* = 4) using the $2^{\Delta\Delta Ct}$ method. *CB*1, cannabinoid receptor type 1 gene (*CNR*[1]; geneID 1268*)*; *CB*[2], cannabinoid receptor type 2 gene (*CNR*[2]; geneID 1269).

Supplementary Table 3: List of genes differnetially expressed following treatment with S4 (5 µg/mL) + S5 (6 µg/mL) only versus control (i.e., these genes were not differentially expressed following S4 or S5 treatment). See Supplementary Table 3

Supplementary Table 4: 947 common genes differentially expressed in both My-La and HuT-78 upon treatment with S4+S5. See Supplementary Table 4

Supplementary Table 5: Values of expression for genes differentially expressed in all treatments (S4, S5 and S4+S5) versus control. See Supplementary Table 5

Supplementary Table 6: Biological pathways with 10 or more genes significantly and at least 2 fold regulated in S4 [5 µg/mL]+S5 [6 µg/mL] treatment versus control in My-La and HuT-78 cell lines. See Supplementary Table 6
